# Understory shrub diversity: equally vital as overstory tree diversity to promote forest productivity

**DOI:** 10.1093/nsr/nwaf093

**Published:** 2025-03-13

**Authors:** Chen Chen, Guoyong Yan, Bernhard Schmid, Yi Li, Franca J Bongers, Helge Bruelheide, Yuanyuan Huang, Shan Li, Goddert von Oheimb, Ting Tang, Kris Verheyen, Bo Yang, Keping Ma, Xiaojuan Liu

**Affiliations:** State Key Laboratory of Forage Breeding-by-Design and Utilization, Key Laboratory of Vegetation and Environmental Change, Institute of Botany, Chinese Academy of Sciences, Beijing 100093, China; China National Botanical Garden, Beijing 100093, China; State Key Laboratory of Forage Breeding-by-Design and Utilization, Key Laboratory of Vegetation and Environmental Change, Institute of Botany, Chinese Academy of Sciences, Beijing 100093, China; School of Life Sciences, Qufu Normal University, Qufu 273165, China; Remote Sensing Laboratories, Department of Geography, University of Zurich, Zurich 8057, Switzerland; State Key Laboratory of Forage Breeding-by-Design and Utilization, Key Laboratory of Vegetation and Environmental Change, Institute of Botany, Chinese Academy of Sciences, Beijing 100093, China; Centre for Crop Systems Analysis, Wageningen University, Wageningen 6700 AK, Netherlands; Institute of Biology/Geobotany and Botanical Garden, Martin Luther University Halle-Wittenberg, Halle 06108, Germany; German Centre for Integrative Biodiversity Research (iDiv) Halle-Jena-Leipzig, Leipzig 04103, Germany; German Centre for Integrative Biodiversity Research (iDiv) Halle-Jena-Leipzig, Leipzig 04103, Germany; Institute of Biology, Leipzig University, Leipzig 04103, Germany; State Key Laboratory of Forage Breeding-by-Design and Utilization, Key Laboratory of Vegetation and Environmental Change, Institute of Botany, Chinese Academy of Sciences, Beijing 100093, China; Institute of General Ecology and Environmental Protection, TUD Dresden University of Technology, Tharandt 01737, Germany; State Key Laboratory of Forage Breeding-by-Design and Utilization, Key Laboratory of Vegetation and Environmental Change, Institute of Botany, Chinese Academy of Sciences, Beijing 100093, China; Forest & Nature Lab, Ghent University, Gontrode 9090, Belgium; School of Biological and Environmental Engineering, Jingdezhen University, Jingdezhen 333032, China; State Key Laboratory of Forage Breeding-by-Design and Utilization, Key Laboratory of Vegetation and Environmental Change, Institute of Botany, Chinese Academy of Sciences, Beijing 100093, China; China National Botanical Garden, Beijing 100093, China; State Key Laboratory of Forage Breeding-by-Design and Utilization, Key Laboratory of Vegetation and Environmental Change, Institute of Botany, Chinese Academy of Sciences, Beijing 100093, China; China National Botanical Garden, Beijing 100093, China; University of Chinese Academy of Sciences, Beijing 100049, China

**Keywords:** biodiversity–ecosystem functioning (BEF), forest productivity, functional diversity, shrub, species richness, stand age

## Abstract

Biodiversity–ecosystem functioning relationships have been extensively studied, particularly within the primary layers of producers in terrestrial ecosystems. In multilayer ecosystems such as forests, the contribution of diversity in the secondary layer, i.e. shrubs, to ecosystem functioning is still largely unknown. Here we used 11-year growth data from a forest biodiversity experiment with factorially crossed manipulations of tree and shrub species richness to assess their effects on forest productivity. We found that shrub species richness had positive effects on tree and total woody biomass (sum of tree and shrub biomass), with effect sizes similar in magnitude to those of tree species richness: increasing tree or shrub species richness from two to eight promoted tree biomass by 73.1% or 53.9% and total woody biomass by 46.7% or 37.1%, respectively. The positive effects of tree or shrub species richness on tree and total woody biomass became larger over time. Shrub biomass was reduced by tree species richness. The effects of tree and shrub species richness can be partially explained by their increased functional diversity. Our study provides the first evidence that understory diversity can significantly increase forest productivity and should not be neglected in forest restoration to promote ecosystem functioning.

## INTRODUCTION

The alarming loss of biodiversity has sparked questions about the relationships between biodiversity and ecosystem functioning (BEF) [1,2]. Over the past three decades, an increasing number of biodiversity experiments simulated biodiversity gradients by planting the same number of individuals but different numbers of species on plots of unit area. These substitutive-design experiments mostly found positive relationships between species number (i.e. richness) and ecosystem functions such as primary productivity [3–6]. In forest biodiversity experiments, studies so far have focused on the species richness of the tree layer [4,7]. However, forests are typically multilayered [8]. The forest understory can harbor rich biodiversity and maintain important ecological functions such as nutrient cycling and supporting soil microorganisms [9–12]. It is conceivable that adding a shrub layer with corresponding changes in species richness would further increase ecosystem functioning, yet we do not know to what extent, or if trade-offs between the two layers might occur, as has been shown, for example, in grassland systems between vascular plants and mosses [13]. Here we ask to what extent understory shrub species richness (hereinafter abbreviated as shrub richness) increases forest productivity, how large this effect is compared to the effect of overstory tree species richness (hereinafter abbreviated as tree richness), how these effects evolve over time, and whether there are trade-offs between the two. Given the inherent relationships between trees and shrubs in natural stands [14–16], answering these questions requires the independent manipulation of both tree and shrub richness and the simultaneous examination of their effects over time.

Both tree and shrub richness may have positive effects on tree biomass. The positive effect of plant diversity on plant biomass has been well documented and is explained by several potential mechanisms [2]. One of the most widely studied mechanisms is that higher tree richness promotes niche complementarity among species with more distinct functional properties [17–20], which reduces competition among trees and consequently increases tree biomass [7,21]. Given the different resource utilization strategies and niches of different shrub species [22], shrub functional diversity may also play an important role in the shrub richness effects on tree biomass, yet this has not been investigated so far. Although shrubs may have different resource utilization strategies from trees, they still compete for vital resources, such as soil nutrients [14,23]. Increased shrub richness and functional diversity may potentially reduce competition of shrubs with trees for some specific nutrients while broadening the overall nutrient competition, leading to both positive and negative effects on tree biomass. Moreover, compared to overstory trees, shrubs are expected to recycle nutrients faster on average and to return a significant amount of nutrients to the soil via high-quality litter [9,24,25]. This characteristic of shrubs may improve the availability of nutrients also for trees, rather than trapping them in slower-reverting pools such as wood and soil organic matter [26]. Since the functional properties of live organs persist through senescence and influence the physical and chemical properties of litter, increasing tree and shrub richness and functional diversity may provide more diverse litter resources for decomposers and enhance nutrient cycling [27–29], thereby potentially promoting the growth of both trees and shrubs. Importantly, increasing tree and shrub diversity can provide more diverse foods and habitats for various trophic levels, including herbivores and predators [30], the diversity of which has shown positive relationships with forest productivity [31]. Considering these possibilities, we assumed that increased shrub richness would have a positive effect on both tree and shrub biomass. Because shrubs are smaller than overstory trees, we also predicted that the effects of higher shrub richness on tree (as well as tree plus shrub) biomass would be less pronounced than the effects of higher tree richness.

Tree and shrub richness may also exert effects on shrub biomass, but in this case, the expected direction of the effects is less clear than for the effects on tree biomass. For understory shrubs, light competition from overstory trees may reduce growth and biomass accumulation [32–34]. With higher tree richness promoting tree biomass and crown complementarity [4,35,36], we predicted that the increased tree biomass would lead to declined shrub biomass in the stands with higher tree richness due to the reduced availability of light and other resources for shrubs. Moreover, based on the positive tree richness–tree biomass relationship in the overstory layer [7,21,37], it can be assumed that shrub richness could have a positive effect on shrub biomass due to greater functional diversity and thus niche partitioning among shrub species [38]. Observations in natural forests have revealed positive or insignificant correlations between shrub richness and shrub biomass [16,39]. However, such observational studies are not able to test the causality of the relationship and may refer to situations where shrub density is low and varies between stands, so the hypothesis cannot be tested effectively. For such a test, experimental manipulation of shrub richness is required, while maintaining shrub density constant, i.e. a substitutive experiment [3,40].

Relationships between species richness and biomass may change over time. The positive effect of species richness on productivity has been shown to increase with time [4,5,7,21]. During the initial development of a planted forest, resources such as light and nutrients may be sufficient for all individuals to grow unconstrained such that increasing richness does not diminish competition and increase biomass accumulation at stand level. As trees grow larger and the canopy closes over time, increased richness can mitigate the increased competition from neighbor trees through niche differentiation [41]. In addition, increased stand age allows for the temporal partitioning of resource use [6] and the accumulation of the species richness effects on soil nutrients and microorganisms [42,43]. In contrast, tree richness may have an increasingly negative effect on shrub growth with increasing stand age, as the positive effect of higher overstory tree richness on tree biomass increases with time. Furthermore, as tree biomass increases and the canopy closes with age, the availability of niches for understory shrubs declines, thereby possibly reducing the importance of shrub species diversity and niche partitioning in promoting shrub biomass in older stands [44]. Therefore, we predicted that the positive effects of tree and shrub richness on tree biomass and the negative effect of tree richness on shrub biomass would become stronger over time, while the effect of shrub richness on shrub biomass would become weaker over time.

**Figure 1.**
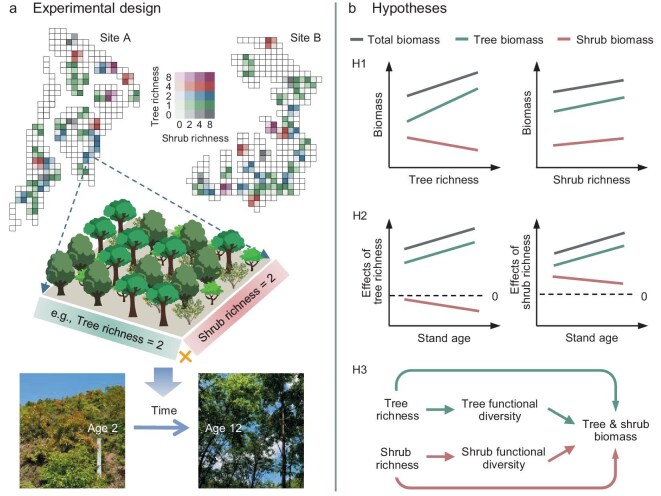
Overview of the hypotheses. (a) Experimental design. (b) Three hypotheses. Experimental design: each plot (25.8 × 25.8 m) was planted with 400 tree seedlings in a square grid design of 20 × 20 individuals with shrub seedlings planted at the middle of four trees; each set of four plots with different shrub richness was randomly arranged in a quadrat with equal tree species composition; the tree and shrub biomass were monitored continuously from stand age 2–12 years. Hypothesis 1: tree species richness (tree richness) promotes tree and total biomass and declines shrub biomass; shrub species richness (shrub richness) promotes tree and shrub biomass and the total woody biomass. Hypothesis 2: the tree and shrub richness effects on tree and total biomass increase over time, while the tree or shrub richness effect on shrub biomass becomes more negative or less positive over time, respectively. Hypothesis 3: tree/shrub richness affects tree/shrub biomass by promoting their functional diversity.

To evaluate the individual and long-term contribution of tree and shrub richness on forest productivity over time, we manipulated both overstory tree richness (0, 1, 2, 4 or 8 tree species) and understory shrub richness (0, 2, 4 or 8 shrub species) in a factorial design in a subtropical forest in southern China (‘BEF-China’ experiment; see Methods; Fig. 1a). We used the aboveground biomass (Mg ha^−1^) of trees and shrubs as the long-term proxy of their productivity, given that trees and shrubs were planted at nearly the same time at our experimental site. We tested the following hypotheses introduced above (Fig. 1b): (i) both tree and shrub richness increase tree biomass and total biomass (sum of tree and shrub biomass), with the effect of shrub richness being smaller than that of tree richness; in contrast, tree richness decreases and shrub richness increases shrub biomass, with the latter effect being smaller; (ii) the positive effect of increasing tree and shrub richness on tree biomass and the negative effect of tree richness on shrub biomass becomes more pronounced with time, while the positive effects of shrub richness on shrub biomass declines; (iii) the effects of tree and shrub richness on tree and shrub biomass are mediated via increased functional diversity of trees and shrubs.

## RESULTS AND DISCUSSION

### Tree and shrub diversity effects on biomass

**Figure 2.**
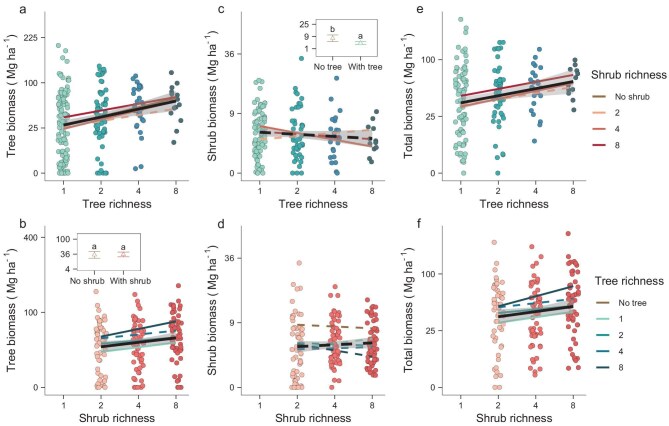
Effects of tree and shrub species richness on the tree, shrub and total woody species biomass at the stand age of 12 years. The lines indicate the variation of biomass along the tree or shrub richness when tested separately within each shrub or tree richness level, respectively, as well as the overall variation of biomass across all shrub or tree richness levels, respectively, with a 95% confidence interval. Significant relationships are shown as solid lines and insignificant ones are shown as dashed lines. The insets in (b) and (c) show the comparison of mean tree and shrub biomass in the plots with and without trees and shrubs, respectively, with a 95% confidence interval. Species richness was natural-logarithm transformed; biomass was square-root transformed.

Our results showed that higher tree richness significantly increased tree biomass (Table 1), with an average increase of 73.1% (standard error, s.e. 30.3%) from a tree richness of two to eight species (Fig. 2a). Also, we showed that increasing shrub richness from two to eight species significantly increased tree biomass by 53.9% (s.e. 26.5%) (Fig. 2b). Planting trees resulted in a significant reduction in shrub biomass compared to control plots without trees, with the effects decreasing with increasing tree richness (Fig. 2c). Shrub richness had an insignificant effect on shrub biomass (Fig. 2d). Total woody plant biomass, i.e. the sum of tree and shrub biomass, increased significantly with tree and shrub richness by 46.7% (s.e. 22.4%) and 37.1% (s.e. 19.6%) from tree and shrub richness of two to eight species, respectively (Fig. 2e–f). These results are consistent with our first hypothesis that an increase in shrub richness promotes tree biomass and total woody biomass. Surprisingly, the positive effect sizes of shrub richness on the tree and total biomass were only 19.2% and 9.6% lower than the corresponding positive effect sizes of tree richness. This indicates that plant diversity within the shrub layer may play a role equally important as that of the tree layer in enhancing forest productivity.

**Table 1.** The effects of tree and shrub species richness, stand age, and their two-way interaction on tree, shrub and total woody species biomass.

	Tree biomass (Mg ha^−1^)				Shrub biomass (Mg ha^−1^)				Total woody biomass (Mg ha^−1^)			
Source of variation	df	ddf	F value	*P*	df	ddf	F value	*P*	df	ddf	F value	*P*
Model 1 (age 12)												
Tree richness	1	167	14.75	**<0.001** 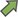	1	165	2.59	0.109	1	164	10.44	**0.001** 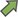
Shrub richness	1	152	5.13	**0.025** 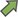	1	155	0.67	0.413	1	150	4.49	**0.036** 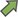
Tree richness × Shrub richness	1	152	<0.01	0.994	1	152	1.19	0.278	1	148	<0.01	0.964
Model 2 (age 2–12)												
Tree richness	1	78	26.51	**<0.001** 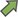	1	59	3.49	0.067 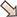	1	79	18.13	**<0.001** 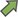
Shrub richness	1	89	6.58	**0.012** 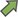	1	119	0.07	0.785	1	72	5.15	**0.026** 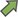
Age	1	157	592.49	**<0.001** 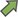	1	149	530.11	**<0.001** 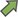	1	155	917.04	**<0.001** 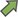
Tree richness × Shrub richness	1	1902	0.65	0.422	1	1577	1.90	0.169	1	1664	0.86	0.353
Tree richness × Age	1	74	26.92	**<0.001** 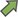	1	58	5.28	**0.025** 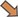	1	75	20.27	**<0.001** 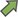
Shrub richness × Age	1	91	5.08	**0.027** 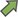	1	123	0.18	0.669	1	75	4.13	**0.046** 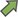

Model 1 examined the effects of tree and shrub richness and their interactions on the biomass at the stand age of 12 years; model 2 added the term stand age and its interaction with tree and shrub richness based on the data across ages of 2–12 years. The analyses were based on linear mixed-effect models fitted by restricted maximum likelihood. Explanations: df, numerator degrees of freedom; ddf, denominator degrees of freedom; F value, F ratio; *P, P* value of the significance test with significant ones (*P* < 0.05) highlighted as bold. Tree richness and Shrub richness are tree and shrub species richness, respectively; upward and downward pointing arrows indicate significant positive and negative effects, respectively.

We found that the effects of tree and shrub richness on tree biomass were independent of each other (interaction effects not significant, Table 1), with the effects of shrub richness on tree biomass being consistently positive irrespective of tree richness (Fig. 2e). The independence of the effects of tree and shrub richness may suggest that they operate via separate mechanisms that do not interfere with each other. While light-use partitioning likely occurs in both layers, the tree overstory is not shaded by the shrubs and the shrubs may just partition the light that is left by the trees. Furthermore, trees and shrubs may have their own independent associations with higher trophic level organisms [45] and decomposers facilitating nutrient cycling [9,24]. Within a single plant layer, the benefits of adding a species are usually less pronounced at high richness compared to additions at low richness, resulting in diminishing returns and flattening response curves of biomass, leading to linear relationships when using log-richness as an independent variable on the x-axis [1,4]. Our results suggest that in a multilayered forest with high tree richness and low shrub richness, adding a shrub species will lead to a greater increase in tree biomass and total woody species biomass than adding a tree species. Given the often large species pool of understory shrubs [11,25], the shrub layer should not be neglected in forest restoration aimed at promoting forest biodiversity and ecosystem functioning.

Shrubs are known to influence forest establishment and succession by competing for resources and by facilitating microenvironments [12,46]. In our experiment, the initial set-up with 0.91 m spacing between trees and shrubs, combined with the relatively young age of the forest (12 years old in the last year of our study period) and the faster growth of most tree species (e.g. greater mean basal diameter than shrub species, see Table S1), likely limited the ability of shrubs to outcompete neighboring trees. Nevertheless, we recognize that the shrub presence, richness and composition, along with various environmental factors, could still exert species-specific effects on trees and, consequently, on forest establishment. For instance, the relatively smaller basal diameter of *Machilus grijsii* compared to some shrubs (Table S1) may indicate potential neighborhood competition or environmental stress, which requires further examination. We encourage more detailed and long-term investigation into shrub–tree interactions across a gradient of stand age and environmental conditions to deepen the understanding of their contribution to forest development.

The insignificant effect of shrub richness on shrub biomass (Table 1) contrasts with previous observational studies that found positive relationships between shrub richness and biomass in naturally occurring shrub communities [16,47]. This contrast may result from the inherent differences between our tree and shrub diversity manipulation experiment and natural forests. The stronger relationships between shrub richness and biomass in natural shrub communities are partially due to their covariation along abiotic or biotic factors, but this covariation was controlled by manipulating shrub richness in our experiment. Additionally, the shrub species pools in our experiment differ from those in previous observational studies. Observational studies usually distinguish between shrubs in the understory and trees in the overstory by size, e.g. by defining shrubs as all woody plants below 1.3 m in height [16,47], which includes seedlings and saplings of tree species in the overstory. In contrast, our experiment planted shrub species that did not overlap with the overstory tree species. Furthermore, in natural forests, shrub density (defined by size) is higher than overstory tree density due to the high mortality rate of seedlings that develop into mature trees [48], whereas in our experiments, shrub individuals had the same spacing as tree individuals. The higher shrub density in natural forests could amplify the effects of shrub diversity on shrub growth by reducing competition among conspecifics.

### Tree and shrub diversity effects across ages

**Figure 3.**
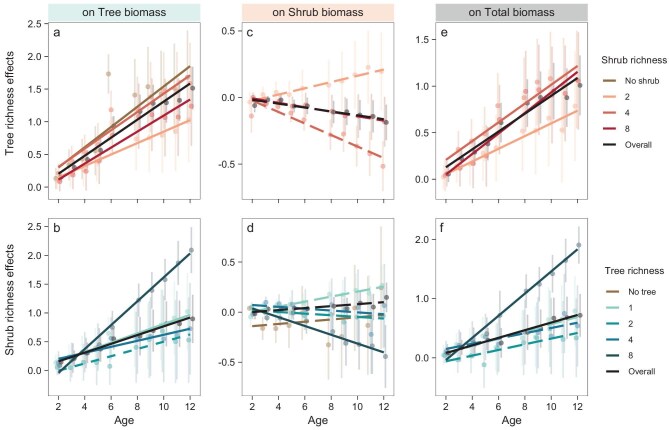
The effects of tree and shrub richness on tree biomass, shrub biomass and total woody species biomass along stand age. The tree and shrub richness effects are indicated by the coefficients and standard errors of their effects on tree, shrub and total biomass in each year using linear mixed-effects models. Meta-regression models were employed to examine the relationships of the tree and shrub richness effects along stand age while accounting for standard errors. Solid regression lines suggest significant changes (*P* < 0.05) and dashed ones suggest insignificant changes.

Our results revealed significant interactions between tree and shrub richness with stand age on tree biomass (Table 1). The positive effects of tree and shrub richness on tree biomass both became more pronounced with time (Fig. 3a–b). The more pronounced richness effects on biomass over time are consistent with our second hypothesis and may be explained by increasing complementarity effects with increasing stand age [4,6,7,21]. As more nutrients are concentrated in slow-turnover pools during stand development, higher shrub richness can become more important for tree productivity in the long term due to the role of shrubs in accelerating nutrient cycling [9,24,25].

In addition, we found that tree richness had an increasingly negative effect on shrub biomass with stand age (Fig. 3c). With increasing stand age, tree richness effects on tree biomass and crown complementarity become more pronounced [4,35], resulting in stronger competition and lower light availability for shrubs [14,16]. Still, the effect of tree richness on shrub biomass may not be entirely negative, as evidenced by the marginally positive impact of tree richness on shrub biomass when shrub richness is two. This may be explained by the increased canopy cover induced by greater tree richness in older stands, which creates milder microclimates that facilitate the growth of understory shrubs [49,50]. Also, the effects of tree richness on soil nutrient cycling become more positive over time, likely due to enhanced soil enzyme activities, which may benefit the growth of understory shrubs [27,43]. Thus, the net effects of overstory richness on the understory will depend on the relative strength of the negative effects, such as reduced light availability, versus the positive effects, such as improved nutrient cycling and altered microclimate.

The temporal variation in the effects of shrub richness on shrub biomass vary across different levels of tree richness, for example, showing a positive trend at the tree richness of one and a declining trend at the tree richness of eight (Fig. 3d). These results partially support our second hypothesis, indicating that the effects of shrub richness on shrub biomass are significantly influenced by the overstory trees. Higher shrub richness may enhance shrub growth through stronger niche complementarity, particularly in tree monocultures where limited crown complementarity creates understory conditions with greater resource niche availability and environmental heterogeneity. Also, since the effect of shrub richness on tree biomass increases particularly strongly with stand age at the tree richness of eight (Fig. 3b), the increased tree biomass may negatively feedback on understory shrubs, leading to decreased shrub biomass. We also showed that the total woody species biomass, similar to tree biomass, was increasingly promoted by tree and shrub richness over time (Fig. 3e–f), because total woody species biomass was mainly composed of tree biomass.

### Tree and shrub functional diversity promotes tree biomass

**Figure 4.**
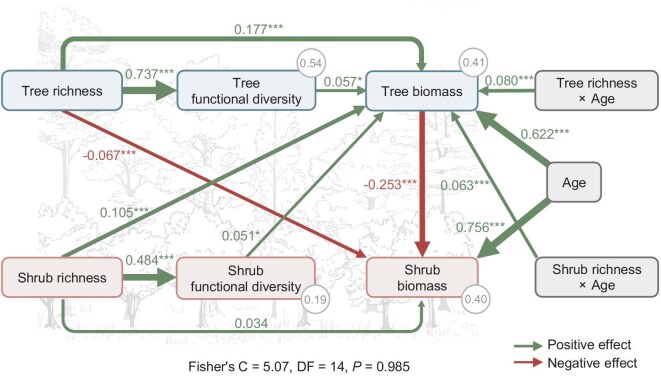
Direct and indirect effects of tree and shrub richness on tree and shrub biomass. The SEM/SCM (see Siegel and Dee, 2025) tests the direct effects of tree and shrub richness as well as their indirect effects mediated via tree and shrub functional diversity on tree and shrub biomass. Data are based on a long-term, experimental species richness gradient with mixtures of 1, 2, 4 and 8 tree species and mixtures of 2, 4 and 8 shrub species. All relationships are significant (*P* < 0.05) with asterisks indicating significance (*P < 0.05, **P < 0.01 and ***P < 0.001) except for the marginally significant effect of shrub richness on shrub biomass (*P* = 0.061). Standardized path coefficients are shown next to each arrow, and the arrow width is scaled by coefficient size. The variation in tree functional diversity, shrub functional diversity, tree biomass and shrub biomass explained in the model (marginal *R* ^2^) is shown in the corresponding circles. Tree and shrub biomass are square-root transformed. ‘Age’ is the growth age of trees; ‘Tree richness × Age’ and ‘Shrub richness × Age’ indicate their interaction effects. Note that tree and shrub richness were manipulated directly without failure or ambiguity and randomized and replicated, and functional diversity measures were based on the tree and shrub functional traits and their composition at the start of the experiment; thus, only the arrow between tree and shrub biomass could potentially be reversed.

To test the third hypothesis that tree and shrub richness promote their biomass due to higher functional diversity, we employed a structural equation model (SEM), in our case of an experimental study identical to a structural causal model (SCM, terminology of Siegel and Dee, 2025), to disentangle the relationships between tree and shrub richness, functional diversity, biomass and stand age (Fig. 4). Before constructing the SEM, we examined the bivariate relationships between tree and shrub richness, functional diversity and biomass, finding that tree and shrub functional diversity, driven by their respective richness, strongly promoted tree biomass, while shrub biomass declined with higher tree functional diversity and showed no significant relationship with shrub functional diversity (Fig. S1). We also examined the effects of the functional diversity of individual tree and shrub traits and found that the functional diversity of both aboveground traits (such as leaf nutrient content and wood density) and belowground traits (such as root diameter and root tissue density) had positive effects on tree biomass (Fig. S2).

The final SEM fitted well to the data (Fisher's C = 5.07, DF = 14, *P* = 0.985), and explained 41% and 40% of the variation in tree and shrub biomass, respectively (Fig. 4). The SEM showed that both tree and shrub richness had positive direct effects on tree biomass (standard coefficient, *r* = 0.177, *P* < 0.001; *r* = 0.105, *P* < 0.001). Also, tree and shrub functional diversity both directly increased tree biomass (*r* = 0.057, *P* = 0.022; *r* = 0.051, *P* = 0.025), leading to positive indirect effects of tree and shrub richness on tree biomass via promoting tree and shrub functional diversity (*r* = 0.042 and 0.025, respectively).

These results are in line with our hypothesis that tree and shrub functional diversity plays an important role in promoting tree biomass. Within the overstory tree layer, increasing tree functional diversity suggests more diverse living strategies and higher niche partitioning, which lowers tree–tree competition and thus benefits tree growth. The effect of shrub functional diversity on tree biomass may operate differently from that of trees. Increasing shrub richness and functional diversity may promote tree biomass via lowering the competition of shrubs for some specific resources with trees [14] and via providing more diverse litter resources that support soil decomposers and their functioning [27,28]. Together with previous studies [7,21], our results suggest that plant functional diversity plays a key role in promoting ecosystem functioning across strata, but also that other mechanisms besides functional diversity-related niche partitioning must be important, for example, species-richness-related promotion of multitrophic interactions [31].

Further, the SEM showed that tree biomass had a strong direct negative effect on shrub biomass (*r* = –0.253, *P* < 0.001). Consistent with observational studies in natural forests [16], our results demonstrate that tree biomass exerts strong competition effects on shrub biomass. In contrast, more than a decade after planting, the observed shrub richness remained aligned with the initial planted richness in the corresponding plots (Fig. S3). This may suggest that while the growth is suppressed, many shrubs can still survive under the intense competitive pressure from trees with distinct survival strategies [11], in addition to the contribution of weeding campaigns to reduce all not-planted plants in our experiment. Our result is in line with the previous observational study showing that shrub biomass but not shrub richness shared a negative relationship with tree biomass in natural forests [16]. We recommend further studies to explore how variations in overstory tree richness influence different shrub species, which shapes understory shrub composition, diversity and productivity. Also, tree richness exerted a direct negative effect on shrub biomass (*r* = –0.067, *P* = 0.001). This implies that tree richness also decreases shrub biomass through other pathways, e.g. the higher crown complementarity reducing light availability for understory shrubs [36]. The SEM also showed that both tree and shrub biomass increased strongly with stand age (*r* = 0.622 and 0.756, respectively; both *P* < 0.001). Increasing stand age promoted the positive effect of tree and shrub richness on tree biomass (*r* = 0.080 and 0.063, respectively; both *P* < 0.001) (Fig. 4), further illustrating the increasingly strong effects of richness on forest biomass. It could be argued that with regard to the relationships between tree and shrub biomass our SEMs only explore hypothetical causal relationships based on correlation. In this case, we rely on biological logic and reasoning [51], which suggests that it is more likely that trees negatively affect shrubs instead of the other way round.

In summary, in comparison with previous studies investigating BEF relationships in primary layers of producers [3–6], our study provides new insights into the BEF relationship in more complex, multilayer systems, and highlights the critical role of understory shrub layer richness in maintaining overall ecosystem functioning. The factorially crossed manipulations of tree and shrub species richness provide the opportunity to examine and compare the independent contributions of tree and shrub richness to forest ecosystem functioning, which cannot be effectively tested in natural stands [16]. Our results reveal surprisingly strong positive effects of shrub richness on tree and total woody species biomass, comparable in relative magnitude to the effects of tree richness. Furthermore, the positive effects of tree and shrub richness became more pronounced with increasing stand age. However, shrub biomass was reduced by tree richness, indicating a slight trade-off of the tree richness effects in the two layers. We also show that the effects of tree and shrub richness can be partially explained by increasing functional diversity, suggesting a key role for niche partitioning among species. As restoration and reforestation projects have been initiated worldwide to restore forest multifunctionality [33], our study suggests that promoting understory shrub richness has the potential to contribute to the multiple goals of promoting forest productivity and carbon storage as well as conserving biodiversity.

## METHODS

### Study site and experimental design

This study was conducted on the BEF-China experimental platform (BEF-China, www.bef-china.com) at Xingangshan, Jiangxi Province, China (29°05–29°08′N, 117°54′–117°56′E), a long-term biodiversity experiment designed to test the effects of tree and shrub richness and composition on forest ecosystem functioning [4,52]. The region is typical for the Chinese subtropics, with a mean annual temperature and precipitation of 16.7°C and 1821 mm, respectively [53]. Detailed information on soil properties in the region can be found in Scholten *et al*. (2017) [54].

A large-scale biodiversity experiment was established by planting seedlings in 2009–2010 at two sites (A and B) on a total area of ∼40 ha. At each site, the species pool comprised 24 broadleaved tree species and 10 shrub species, with 8 tree species and 2 shrub species overlapping between the two sites (Table S1). We used these pools to create different extinction scenarios to study the diversity loss effects on ecosystem functioning and to achieve a high variation in species compositions across the species richness levels (see details in Huang *et al*. (2018) [4], Yang *et al*. (2013) [53] and Bruelheide *et al*. (2014) [52]). We first randomly selected three minimally overlapping pools of 16 tree species from a set of 24 species per site and randomly split each of the 6 species pools repeatedly into halves, resulting in nested, non-overlapping subsets of 8, 4, 2 and 1 tree species. This ensures that the tree species composition of communities at the same richness level does not overlap within species pools and that all species occur equally across all richness levels. For studying the tree richness and shrub richness effects, this study was based on a subset of plots (264 plots) in which both tree richness (0, 1, 2, 4, 8) and shrub richness (0, 2, 4, 8) were manipulated (Fig. 1a). The shrub richness treatments were applied to a larger quadrat of four plots with the same tree species composition, planting 0, 2, 4 or 8 shrub species in each plot (Fig. 1a). The species compositions of shrubs were determined using a random partition design from the pool of 10 shrub species at each site (Tables S1–S3). The basic plot size in BEF-China is 25.8 × 25.8 m, which corresponds to 1/15 ha, the Chinese unit area of 1 mu. Each plot was planted with 400 tree seedlings in a square grid of 20 × 20 individuals. The shrubs were planted in the center between four trees. The spacing between the two closest tree (or shrub) individuals was 1.29 m, while the spacing between the closest tree and shrub was 0.91 m. In addition, the shrub species were also planted alone without trees, with only 0, 2, 4 or 8 shrub species in 1-mu plots. Shrubs were planted one year later than the trees. In this study, we defined stand age as the year of tree plantation and used biomass data for trees and shrubs starting from the second year, when shrubs were planted. To maintain the designed tree and shrub composition in each plot as well as possible, herbaceous and non-planted woody species were removed in weeding campaigns once per year [52].

### Tree and shrub measurements

We studied how the change in tree and shrub richness along the simulated extinction scenarios affected the accumulated biomass of trees, shrubs and their sum (= total woody species biomass) from 2011–2022. The diameter at breast height (DBH) and height of all surviving trees of the 16 central trees in each plot of 400 trees per site per year were measured. Following Huang *et al*. (2018) [4], we used the same allometry equation for all tree species to estimate each tree's aboveground biomass using tree height and DBH as predictors to avoid artifacts due to species effects. We measured the height and basal diameter of all surviving shrubs of 5 × 5 individuals at the center of the 1 mu plot at the tree richness levels of 1 and 2, and 11 × 11 individuals at the center of the 1 mu plot at the tree richness levels of 4 and 8. For plots planted with shrubs but not trees, 6 × 6 shrub individuals were measured at the center of the plots with shrub richness of 2, and 12 × 12 individuals were measured at the center of the plots with shrub richness of 4 and 8. Similar to the generalized allometric models for global shrubs [55], we calculated the biomass for each surviving shrub based on basal area and height using the same allometric equation [56]. The total woody species biomass was calculated as the sum of tree and shrub biomass.

### Functional diversity estimation

The tree and shrub functional diversity in each plot was calculated as functional dispersion based on the tree and shrub functional traits and their composition at the start of the experiment using the ‘FD’ package in R [57]. Based on the experimental design, tree and shrub species were planted with equal proportions in the plots with two or more species. The species-level tree functional traits were measured in 2020, 2021 and 2023. Tree functional traits were measured following previous studies [58–60], including leaf habit: deciduous vs. evergreen; leaf structural traits: specific leaf area (the one-sided area of a fresh leaf divided by its oven-dry mass, expressed in cm^2^ g^−1^), leaf dry matter content (leaf oven-dry mass divided by the fresh mass, expressed in mg g^−1^), leaf thickness (mm), stomatal area (the area of a stomata on the leaf, expressed in μm^2^) and stomatal density (stomatal number per unit leaf area; expressed in mm^−2^); leaf chemical traits: leaf nitrogen content (mg g^−1^), leaf phosphorus content (mg g^−1^), leaf potassium content (mg g^−1^) and leaf chlorophyll content (μg cm^−2^); stem traits: number of branches and wood density (wood dry mass per unit of fresh volume, expressed in g cm^−3^); root traits: root diameter (mm), specific root length (root length per dry mass, expressed in m g^−1^) and root tissue density (root dry mass per root volume, expressed in g cm^−3^) of absorbing roots (1–3 orders); and mycorrhizal associations (ectomycorrhizal or arbuscular mycorrhizal associations). The species-level shrub functional traits were measured in 2021. Shrub functional traits included: leaf habit: deciduous vs. evergreen; leaf structural traits: specific leaf area (cm^2^ g^−1^), leaf dry matter content (mg g^−1^), leaf thickness (mm), stomatal area (μm^2^) and stomatal density (mm^−2^); leaf chemical traits: leaf nitrogen content (mg g^−1^) and leaf phosphorus content (mg g^−1^); stem traits: wood density (g cm^−3^), maximum water conductivity (mass of flow through perfusion solution through the piece of wood per unit of time and cross-sectional area of the twig, expressed in kg m^−1^ s^−1^ MPa^−1^) and stem growth rate (cm^2^ yr^−1^); root traits: root diameter (mm), specific root length (m g^−1^) and root tissue density (g cm^−3^) of absorbing roots (1–3 orders).

### Statistical analyses

First, to test hypothesis 1, we examined the tree and shrub richness effects on tree, shrub and total woody biomass at the stand age of 12 years, which was the most recent measurement of tree and shrub biomass. We employed a linear mixed-effects model with tree and shrub richness and their two-way interaction as fixed effects:


(1) \begin{eqnarray*}
\textit{Biomass} &=& {\beta _0} + {\beta _1} \cdot \textit{Tree}\,\textit{richness} \\
&&\!\!+\, {\beta _2} \cdot \textit{Shrub}\,\textit{richness} + {\beta _3} \cdot \textit{Tree}\,\textit{richness} \\
&&\!\!\times \textit{Shrub}\,\textit{richness}\! +\! {\pi _{\textit{site}}}\! +\! {\pi _{\textit{condition}}}\! +\! \varepsilon\\
\end{eqnarray*}

where *Biomass* is the stand-level tree, shrub or total woody biomass and *ε* is the sampling error. We included a random-effects term of plot condition, *π_condition_*, which was composed of altitude, slope direction, slope degree and solar radiation of each plot, to account for the heterogeneity of topography within each experimental site (A and B). The altitude was classified into four levels: 100–150 m, 150–200 m, 200–250 m and 250–300 m; slope direction was classified into three categories: facing north, south and middle; the slope degrees were classified into three categories: flat (0–15°), middle (15–30°) and steep (30–45°); the solar radiation was classified into three categories based on the solar energy per unit area (kWh m^−^²): low, medium and high. We used this random-effects term of plot condition instead of the identity (ID) of the four-plot quadrats with equal tree species composition due to the topographical differences within the quadrat. However, we also examined models in which we added the ID of the four-plot quadrats as a random-effects term (Table S4), which yielded results similar to those without it (Table 1). The tree richness gradient included richness of 1, 2, 4, 8, and the shrub richness gradient included richness of 2, 4, 8. The biomass was square-root transformed to increase the normality and homoscedasticity of residuals, while tree or shrub richness was natural-logarithm transformed to linearize relationships.

To test hypothesis 2, we further examined the effects of stand age and its two-way interaction with tree and shrub richness in addition to the richness effects, based on the data set with continuous measurements of tree and shrub biomass during the ages of 2–12 years. Based on Equation 1, we added stand age as a fixed effect term in the linear mixed effect model:


(2) \begin{eqnarray*}
\textit{Biomass} &=& {\beta _0} + {\beta _1} \cdot \textit{Tree}\,\textit{richness} \\
&& +\, {\beta _2}\cdot \textit{Shrub}\,\textit{richness} + {\beta _3} \cdot Age \\
&& +\, {\beta _4}\cdot \textit{Tree}\,\textit{richness} \times \textit{Shrub}\,\textit{richness}\\
&& +\, {\beta _5} \cdot \textit{Tree}\,\textit{richness} \times Age\\
&& +\, {\beta _6} \cdot \textit{Shrub}\,\textit{richness} \times Age + {\pi _{\textit{site}}}\\
&& +\, {\pi _{\textit{condition}}} + {\pi _{\textit{plot}}} + \varepsilon
\end{eqnarray*}

where *Age* is stand age; *π_plot_* is the random factor of 1 mu plot identity accounting for the repeated-measures design. Note that *Age* = 1 is 2009 for site A and *Age* = 1 is 2010 for site B. The linear mixed-effects models were fitted by restricted maximum likelihood using the ‘lme4’ package [61]. To further demonstrate the change in richness effects over time, we fitted linear mixed-effects models for the biomass along the variation of tree or shrub richness at each level of shrub or tree richness, respectively, at each stand age, while accounting for the random effects of site and plot condition. We extracted the coefficients of tree and shrub richness as their effect sizes and their standard errors, and examined the variation of these effect sizes with stand age using meta-regression models [62]. This meta-regression approach, for example as used in Bongers *et al.* (2021) [21], has the advantage that each coefficient is evaluated using its own error variation, whereas the overall analysis fits a common error variation for all coefficients.

To test hypothesis 3, we employed a SEM to examine the effects of tree and shrub richness on biomass via tree and shrub functional diversity. The SEM modeling started with all potential variables, including tree and shrub richness, functional diversity and biomass, as well as stand age, and the interaction effects of stand age with tree and shrub richness, with the paths indicated by hypothesis 3 and all other plausible paths (Fig. S4). We implemented the SEM using the package ‘piecewiseSEM’ in R [63]. The final model was selected based on Fisher's C statistic (*P* > 0.05 for a satisfactory fit) and the Akaike information criterion (AIC) values [63]. Note that our SEMs in the terminology of Siegel and Dee (2025) correspond to SCMs [64], because they were derived from an experimental study where tree and shrub richness were manipulated directly without failure or ambiguity and randomized and replicated, functional diversity measures were based on the tree and shrub functional traits and their composition at the start of the experiment, and age obviously could not be affected by other variables. All statistical analyses were conducted in R 4.3.3 [65].

## Supplementary Material

nwaf093_Supplemental_File

## Data Availability

The data supporting the findings of this study are presented in the figures. Raw data sets generated during the research are available from the corresponding author upon reasonable request.
