# Supplementary material for: Understory shrub diversity: equally vital as overstory tree diversity to promote forest productivity
Source: Natl Sci Rev. 2025 Mar 13;12(7):nwaf093. doi: 10.1093/nsr/nwaf093 (PMC12236163; doi:10.1093/nsr/nwaf093)
Supplement: nwaf093_Supplemental_File [file nwaf093_supplemental_file.docx]

# Supplementary information for:

**Understory shrub diversity: equally vital as overstory tree diversity to promote forest productivity**

Chen Chen, Guoyong Yan, Bernhard Schmid, Yi Li, Franca J. Bongers, Helge Bruelheide, Yuanyuan Huang, Shan Li, Goddert von Oheimb, Ting Tang, Kris Verheyen, Bo Yang, Keping Ma, Xiaojuan Liu*

## Table S1. Tree and shrub species included in the BEF-China experiment in the last year of the studied period.

| **Lifeform** | **Species No.** | **Species** | **Family** | **Site** | **Leaf duration** | **Mean basal diameter (cm)** |
| --- | --- | --- | --- | --- | --- | --- |
| Tree | 1 | *Acer davidii* Franch. | Aceraceae | A | Deciduous | 6.66 |
|  | 2 | *Ailanthus altissima* (Mill.) Swingle | Simaroubaceae | B | Deciduous | 7.77 |
|  | 3 | *Alniphyllum fortunei* (Hemsl.) Makino | Styracaceae | B | Deciduous | 10.62 |
|  | 4 | *Betula luminifera* H. Winkl. | Betulaceae | B | Deciduous | 6.99 |
|  | 5 | *Castanea henryi* (Skan) Rehd. et Wils. | Fagaceae | A | Deciduous | 12.15 |
|  | 6 | *Castanopsis carlesii* (Hemsl.) Hayata. | Fagaceae | A | Evergreen | 5.80 |
|  | 7 | *Castanopsis eyrei* (Champ. ex Benth.) Tutch. | Fagaceae | A&B | Evergreen | 10.05 |
|  | 8 | *Castanopsis fargesii* Franch. | Fagaceae | B | Evergreen | 12.37 |
|  | 9 | *Castanopsis sclerophylla* (Lindl. et Paxton) Schottky | Fagaceae | A&B | Evergreen | 6.93 |
|  | 10 | *Celtis biondii* Pamp. | Ulmaceae | B | Deciduous | 4.70 |
|  | 11 | *Choerospondias axillaris* (Roxb.) B.L.Burtt & A.W.Hill | Anacardiaceae | A | Deciduous | 13.99 |
|  | 12 | *Cinnamomum camphora* (L.) Presl | Lauraceae | A&B | Evergreen | 9.28 |
|  | 13 | *Cyclobalanopsis glauca* (Thunberg) Oersted | Fagaceae | A&B | Evergreen | 7.68 |
|  | 14 | *Cyclobalanopsis myrsinifolia* (Blume) Oersted | Fagaceae | A | Evergreen | 10.89 |
|  | 15 | *Daphniphyllum oldhamii* (Hemsl.) Rosenthal | Daphniphyllaceae | A&B | Evergreen | 12.08 |
|  | 16 | *Diospyros japonica* Siebold & Zuccarini | Ebenaceae | A&B | Deciduous | 5.50 |
|  | 17 | *Elaeocarpus chinensis* (Gardn. et Chanp.) Hook. f. ex Benth. | Elaeocarpaceae | B | Evergreen | 12.01 |
|  | 18 | *Elaeocarpus glabripetalus* Merr. | Elaeocarpaceae | B | Evergreen | 11.49 |
|  | 19 | *Elaeocarpus japonicus* Sieb. et Zucc. | Elaeocarpaceae | B | Evergreen | 13.71 |
|  | 20 | *Idesia polycarpa* Maxim. | Flacourtiaceae | B | Deciduous | 7.96 |
|  | 21 | *Koelreuteria bipinnata* Franch. | Sapindaceae | A | Deciduous | 5.69 |
|  | 22 | *Liquidambar formosana* Hance | Hamamelidaceae | A | Deciduous | 10.00 |
|  | 23 | *Lithocarpus glaber* (Thunb.) Nakai | Fagaceae | A&B | Evergreen | 8.83 |
|  | 24 | *Machilus grijsii* Hance | Lauraceae | B | Evergreen | 2.76 |
|  | 25 | *Machilus leptophylla* Hand.-Mazz. | Lauraceae | B | Evergreen | 5.67 |
|  | 26 | *Machilus thunbergii* Sieb. et Zucc. | Lauraceae | B | Evergreen | 6.97 |
|  | 27 | *Manglietia fordiana* Oliv. | Magnoliaceae | B | Evergreen | 9.97 |
|  | 28 | *Melia azedarach* L. | Meliaceae | A | Deciduous | 7.88 |
|  | 29 | *Meliosma flexuosa* Pamp. | Sabiaceae | B | Deciduous | 7.76 |
|  | 30 | *Nyssa sinensis* Oliv. | Nyssaceae | A | Deciduous | 11.45 |
|  | 31 | *Phoebe bournei* (Hemsl.) Yang | Lauraceae | B | Evergreen | 6.49 |
|  | 32 | *Quercus acutissima* Carr. | Fagaceae | A | Deciduous | 10.20 |
|  | 33 | *Quercus fabri* Hance | Fagaceae | A | Deciduous | 5.63 |
|  | 34 | *Quercus phillyreoides* A. Gray | Fagaceae | B | Evergreen | 4.42 |
|  | 35 | *Quercus serrata* Murray | Fagaceae | A | Deciduous | 5.22 |
|  | 36 | *Rhus chinensis* Mill. | Anacardiaceae | A | Deciduous | 4.85 |
|  | 37 | *Sapindus saponaria* Linnaeus | Sapindaceae | A | Deciduous | 6.25 |
|  | 38 | *Schima superba* Gardn. et Champ. | Theaceae | A&B | Evergreen | 15.15 |
|  | 39 | *Triadica cochinchinensis* Loureiro | Euphorbiaceae | A | Deciduous | 15.17 |
|  | 40 | *Triadica sebifera* (Linnaeus) Small | Euphorbiaceae | A | Deciduous | 6.90 |
| Shrub | 1 | *Ardisia crenata* Sims | Myrsinaceae | A | Evergreen | 0.85 |
|  | 2 | *Camellia chekiangoleosa* Hu | Theaceae | A | Evergreen | 2.04 |
|  | 3 | *Distylium buxifolium* (Hance) Merr. | Hamamelidaceae | A&B | Evergreen | 4.15 |
|  | 4 | *Distylium myricoides* Hemsl. | Hamamelidaceae | A | Evergreen | 3.90 |
|  | 5 | *Euonymus myrianthus* Hemsl. | Celastraceae | A | Evergreen | 0.59 |
|  | 6 | *Eurya muricata* Dunn | Theaceae | A | Evergreen | 4.08 |
|  | 7 | *Ficus erecta* Thunb. | Moraceae | B | Deciduous | 0.63 |
|  | 8 | *Gardenia jasminoides* Ellis | Rubiaceae | A | Evergreen | 1.49 |
|  | 9 | *Hydrangea chinensis* Maxim. | Saxifragaceae | B | Deciduous | 0.80 |
|  | 10 | *Itea omeiensis* C. K. Schneider | Saxifragaceae | B | Evergreen | 1.97 |
|  | 11 | *Loropetalum chinense* (R. Br.) Oliver | Hamamelidaceae | A | Evergreen | 1.87 |
|  | 12 | *Photinia hirsuta* Hand.-Mazz. | Rosaceae | B | Deciduous | 1.48 |
|  | 13 | *Phyllanthus glaucus* Wall. ex Muell. Arg | Euphorbiaceae | B | Deciduous | 2.10 |
|  | 14 | *Rhaphiolepis indica* (Linnaeus) Lindley | Rosaceae | A | Evergreen | 0.89 |
|  | 15 | *Rhododendron ovatum* (Lindl.) Planch. | Ericaceae | B | Evergreen | 2.59 |
|  | 16 | *Rhododendron simsii* Planch. | Ericaceae | B | Deciduous | 1.05 |
|  | 17 | *Syzygium buxifolium* Hook. et Arn. | Myrtaceae | A&B | Evergreen | 3.50 |
|  | 18 | *Viburnum setigerum* Hance | Adoxaceae | B | Deciduous | 2.00 |

## Table S2. The composition of the eight-species shrub mixture at Site A. Two-species and four-species shrub mixtures were created by randomly selecting species from the corresponding eight-species mixture. “1” and “0” indicate the presence and absence of a species, respectively. The name of the shrub species corresponding to each species number is provided in Table S1.

| 8-species mixture no. | Shrub species no. | | | | | | | | | | |
| --- | --- | --- | --- | --- | --- | --- | --- | --- | --- | --- | --- |
|  | 1 | 2 | 3 | 4 | 5 | 6 | 8 | 11 | 14 | 17 |  |
| 1 | 1 | 1 | 1 | 1 | 1 | 1 | 1 | 1 | 0 | 0 |  |
| 2 | 1 | 1 | 1 | 1 | 1 | 1 | 0 | 1 | 1 | 0 |  |
| 3 | 1 | 1 | 1 | 1 | 1 | 0 | 1 | 1 | 1 | 0 |  |
| 4 | 1 | 1 | 1 | 1 | 1 | 0 | 1 | 1 | 0 | 1 |  |
| 5 | 1 | 1 | 1 | 1 | 1 | 0 | 1 | 0 | 1 | 1 |  |
| 6 | 1 | 1 | 1 | 1 | 0 | 1 | 1 | 1 | 0 | 1 |  |
| 7 | 1 | 1 | 1 | 1 | 0 | 1 | 1 | 0 | 1 | 1 |  |
| 8 | 1 | 1 | 1 | 1 | 0 | 1 | 0 | 1 | 1 | 1 |  |
| 9 | 1 | 1 | 1 | 1 | 0 | 0 | 1 | 1 | 1 | 1 |  |
| 10 | 1 | 1 | 1 | 0 | 1 | 1 | 1 | 1 | 1 | 0 |  |
| 11 | 1 | 1 | 1 | 0 | 1 | 1 | 1 | 0 | 1 | 1 |  |
| 12 | 1 | 1 | 1 | 0 | 1 | 1 | 0 | 1 | 1 | 1 |  |
| 13 | 1 | 1 | 1 | 0 | 1 | 0 | 1 | 1 | 1 | 1 |  |
| 14 | 1 | 1 | 0 | 1 | 1 | 1 | 1 | 1 | 0 | 1 |  |
| 15 | 1 | 1 | 0 | 1 | 1 | 1 | 0 | 1 | 1 | 1 |  |
| 16 | 1 | 1 | 0 | 1 | 1 | 0 | 1 | 1 | 1 | 1 |  |
| 17 | 1 | 1 | 0 | 0 | 1 | 1 | 1 | 1 | 1 | 1 |  |
| 18 | 1 | 0 | 1 | 1 | 1 | 1 | 1 | 1 | 1 | 0 |  |
| 19 | 1 | 0 | 1 | 1 | 1 | 1 | 1 | 1 | 0 | 1 |  |
| 20 | 1 | 0 | 1 | 1 | 1 | 1 | 1 | 0 | 1 | 1 |  |
| 21 | 1 | 0 | 1 | 1 | 1 | 1 | 0 | 1 | 1 | 1 |  |
| 22 | 1 | 0 | 1 | 1 | 0 | 1 | 1 | 1 | 1 | 1 |  |
| 23 | 0 | 1 | 1 | 1 | 1 | 1 | 1 | 1 | 1 | 0 |  |
| 24 | 0 | 1 | 1 | 1 | 1 | 1 | 1 | 1 | 0 | 1 |  |
| 25 | 0 | 1 | 1 | 1 | 1 | 1 | 1 | 0 | 1 | 1 |  |
| 26 | 0 | 1 | 1 | 1 | 1 | 1 | 0 | 1 | 1 | 1 |  |
| 27 | 0 | 1 | 1 | 0 | 1 | 1 | 1 | 1 | 1 | 1 |  |
| 28 | 0 | 1 | 0 | 1 | 1 | 1 | 1 | 1 | 1 | 1 |  |
| 29 | 0 | 0 | 1 | 1 | 1 | 1 | 1 | 1 | 1 | 1 |  |

## Table S3. The composition of the eight-species shrub mixture at Site B. Two-species and four-species shrub mixtures were created by randomly selecting species from the corresponding eight-species mixture. “1” and “0” indicate the presence and absence of a species, respectively. The name of the shrub species corresponding to each species number is provided in Table S1.

| 8-species mixture no. | Shrub species no. | | | | | | | | | |
| --- | --- | --- | --- | --- | --- | --- | --- | --- | --- | --- |
|  | 3 | 7 | 9 | 10 | 12 | 13 | 15 | 16 | 17 | 18 |
| 1 | 1 | 1 | 1 | 1 | 1 | 1 | 1 | 1 | 0 | 0 |
| 2 | 1 | 1 | 1 | 1 | 1 | 1 | 0 | 1 | 1 | 0 |
| 3 | 1 | 1 | 1 | 1 | 1 | 1 | 0 | 0 | 1 | 1 |
| 4 | 1 | 1 | 1 | 1 | 1 | 0 | 1 | 1 | 1 | 0 |
| 5 | 1 | 1 | 1 | 1 | 1 | 0 | 1 | 1 | 0 | 1 |
| 6 | 1 | 1 | 1 | 1 | 0 | 1 | 1 | 0 | 1 | 1 |
| 7 | 1 | 1 | 1 | 1 | 0 | 1 | 0 | 1 | 1 | 1 |
| 8 | 1 | 1 | 1 | 1 | 0 | 0 | 1 | 1 | 1 | 1 |
| 9 | 1 | 1 | 1 | 0 | 1 | 1 | 0 | 1 | 1 | 1 |
| 10 | 1 | 1 | 1 | 0 | 1 | 0 | 1 | 1 | 1 | 1 |
| 11 | 1 | 1 | 0 | 1 | 1 | 1 | 1 | 1 | 1 | 0 |
| 12 | 1 | 1 | 0 | 1 | 1 | 1 | 1 | 1 | 0 | 1 |
| 13 | 1 | 1 | 0 | 1 | 1 | 1 | 1 | 0 | 1 | 1 |
| 14 | 1 | 1 | 0 | 1 | 1 | 1 | 0 | 1 | 1 | 1 |
| 15 | 1 | 1 | 0 | 1 | 1 | 0 | 1 | 1 | 1 | 1 |
| 16 | 1 | 1 | 0 | 0 | 1 | 1 | 1 | 1 | 1 | 1 |
| 17 | 1 | 0 | 1 | 1 | 1 | 1 | 1 | 1 | 1 | 0 |
| 18 | 1 | 0 | 1 | 1 | 1 | 1 | 1 | 1 | 0 | 1 |
| 19 | 1 | 0 | 1 | 1 | 1 | 1 | 1 | 0 | 1 | 1 |
| 20 | 1 | 0 | 1 | 1 | 1 | 1 | 0 | 1 | 1 | 1 |
| 21 | 1 | 0 | 0 | 1 | 1 | 1 | 1 | 1 | 1 | 1 |
| 22 | 0 | 1 | 1 | 1 | 1 | 1 | 1 | 1 | 0 | 1 |
| 23 | 0 | 1 | 1 | 1 | 1 | 0 | 1 | 1 | 1 | 1 |
| 24 | 0 | 1 | 1 | 1 | 0 | 1 | 1 | 1 | 1 | 1 |
| 25 | 0 | 1 | 1 | 0 | 1 | 1 | 1 | 1 | 1 | 1 |
| 26 | 0 | 0 | 1 | 1 | 1 | 1 | 1 | 1 | 1 | 1 |

## Table S4. The effects of tree and shrub species richness, stand age, and their two-way interaction on tree, shrub, and total woody species biomass while accounting for the random effect of the identity (ID) of the four-plot quadrat with the same tree species composition. The results are based on the linear mixed-effect models shown as Eqn. 1 and 2 with adding the random effects of the ID of four-plot quadrats. Explanations: df, numerator degrees of freedom; ddf, denominator degrees of freedom; F value, F ratio; *P*, *P* value of the significance test with significant ones (*P* < 0.05) highlighted as bold; TreeR and ShrubR are tree and shrub species richness, respectively.

| **Source of variation** | **Tree biomass (Mg ha^-1^)** | | | | **Shrub biomass (Mg ha^-1^)** | | | | **Total woody biomass (Mg ha^-1^)** | | | |
| --- | --- | --- | --- | --- | --- | --- | --- | --- | --- | --- | --- | --- |
|  | **df** | **ddf** | **F value** | ***P*** | **df** | **ddf** | **F value** | ***P*** | **df** | **ddf** | **F value** | ***P*** |
| Model 1 (age 12) | | | | | | | | | | | | |
| TreeR | 1 | 56 | 4.80 | **0.033** | 1 | 54 | 1.03 | 0.314 | 1 | 55 | 3.46 | 0.068 |
| ShrubR | 1 | 111 | 13.41 | **< 0.001** | 1 | 106 | 1.23 | 0.269 | 1 | 107 | 12.41 | **< 0.001** |
| TreeR × ShrubR | 1 | 110 | 0.25 | 0.620 | 1 | 102 | 2.00 | 0.160 | 1 | 106 | 0.46 | 0.500 |
| Model 2 (age 2–12) | | | | | | | | | | | | |
| TreeR | 1 | 109 | 10.00 | **0.002** | 1 | 86 | 2.63 | 0.109 | 1 | 106 | 7.20 | **0.008** |
| ShrubR | 1 | 65 | 7.26 | **0.009** | 1 | 117 | 0.06 | 0.808 | 1 | 54 | 6.04 | **0.017** |
| Age | 1 | 150 | 610.8 | **< 0.001** | 1 | 147 | 466.66 | **< 0.001** | 1 | 145 | 941.31 | **< 0.001** |
| TreeR × ShrubR | 1 | 592 | 1.17 | 0.279 | 1 | 977 | 3.15 | 0.076 | 1 | 638 | 1.32 | 0.251 |
| TreeR × Age | 1 | 97 | 29.90 | **< 0.001** | 1 | 58 | 5.27 | **0.025** | 1 | 76 | 20.53 | **< 0.001** |
| ShrubR × Age | 1 | 66 | 6.85 | **0.011** | 1 | 119 | 0.19 | 0.665 | 1 | 55 | 5.54 | **0.022** |


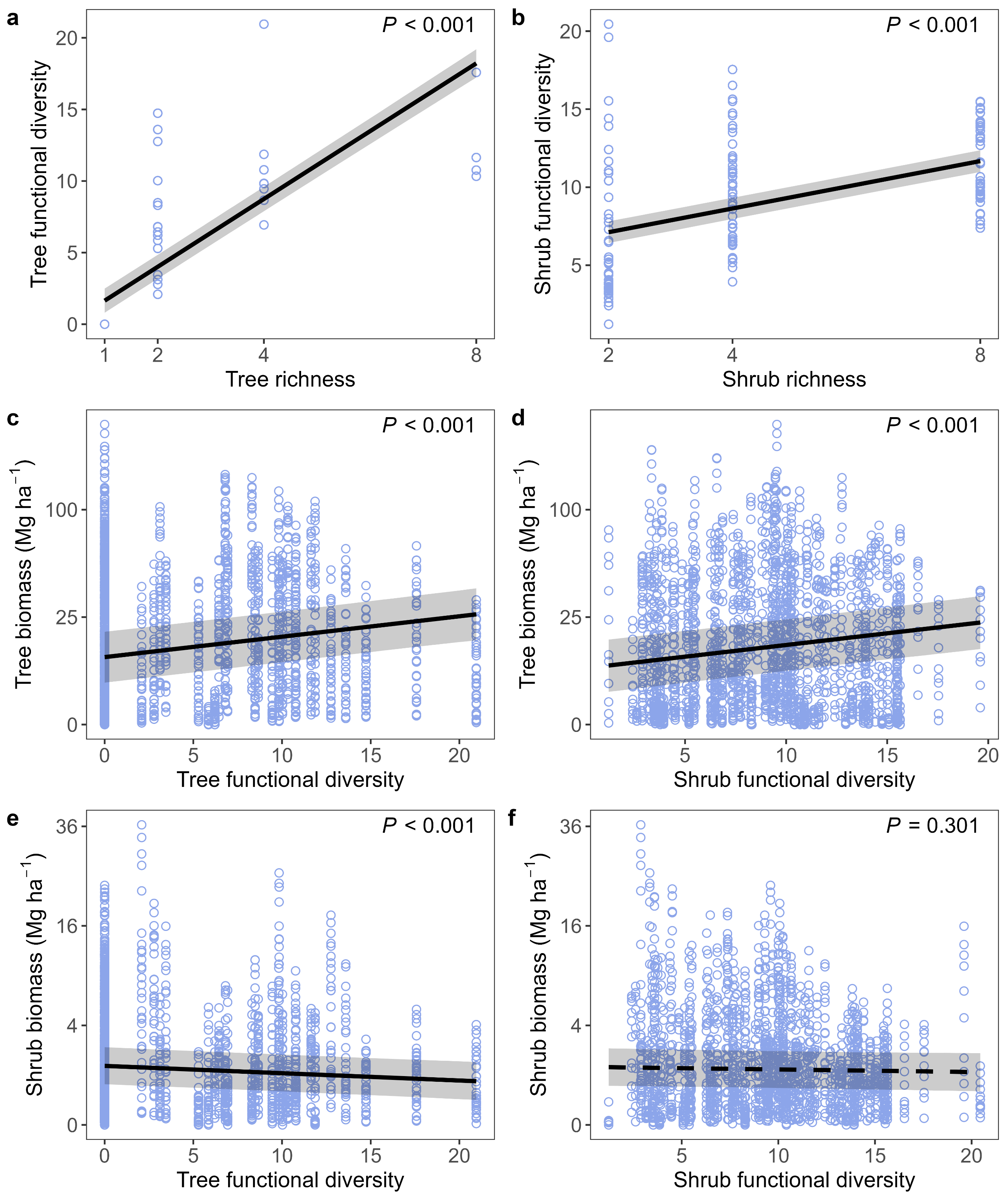


## **Figure S1. The bivariate relationships between tree and shrub species richness, functional diversity, and biomass.** The fitted lines along with the 95% confidence intervals are based on the bivariate relationships between the two variables, analyzed using linear mixed-effects models. Significant and non-significant relationships are indicated with solid and dashed lines, respectively. Data points in the background are observed values.


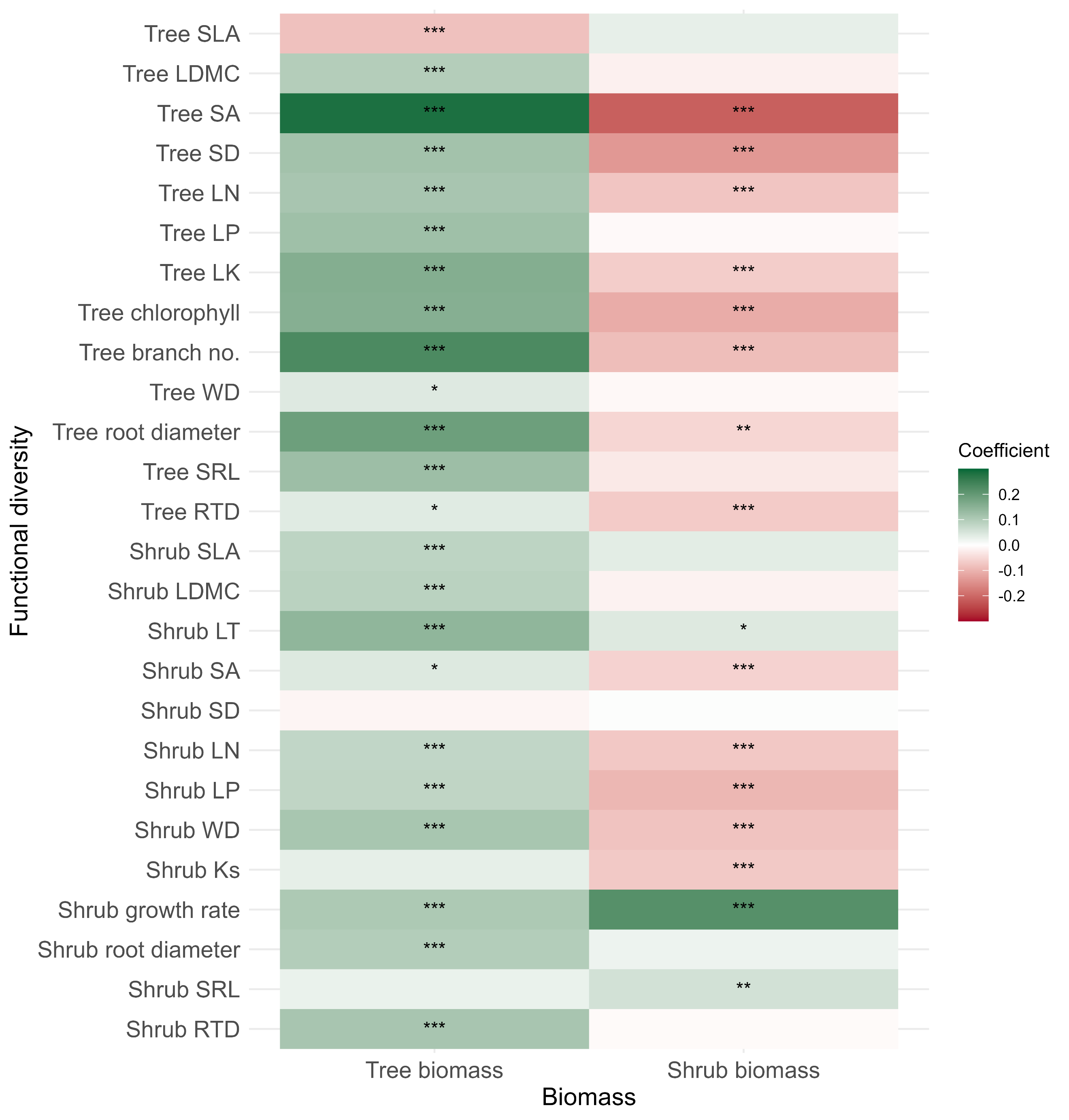


## **Figure S2.** The relationships between tree and shrub biomass with tree and shrub functional diversity. The effect sizes (coefficients) are indicated by the colour gradient. SLA, specific leaf area; LDMC, leaf dry matter content; SA, stomatal area; SD, stomatal density; LN, leaf nitrogen content; LP; leaf phosphorus content; LK, leaf potassium content; WD, wood density; SRL, specific root length; RTD, root tissue density; LT, leaf thickness; Ks, branch stem maximum water conductivity.


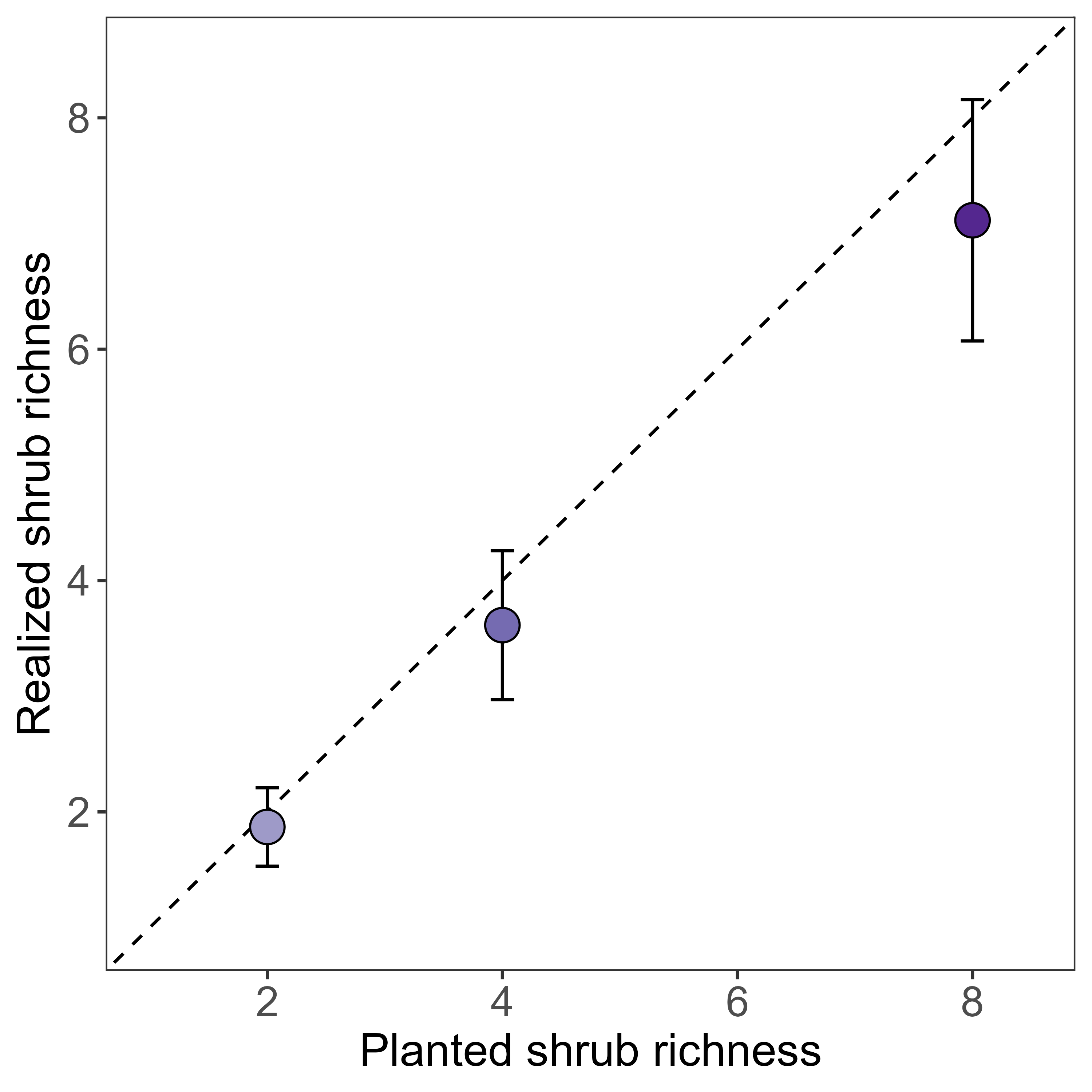


## Figure S3. Realized shrub richness in the most recent whole-plot survey of shrubs in 2024. The mean and the standard deviation of the shrub richness are indicated as points and error bars.


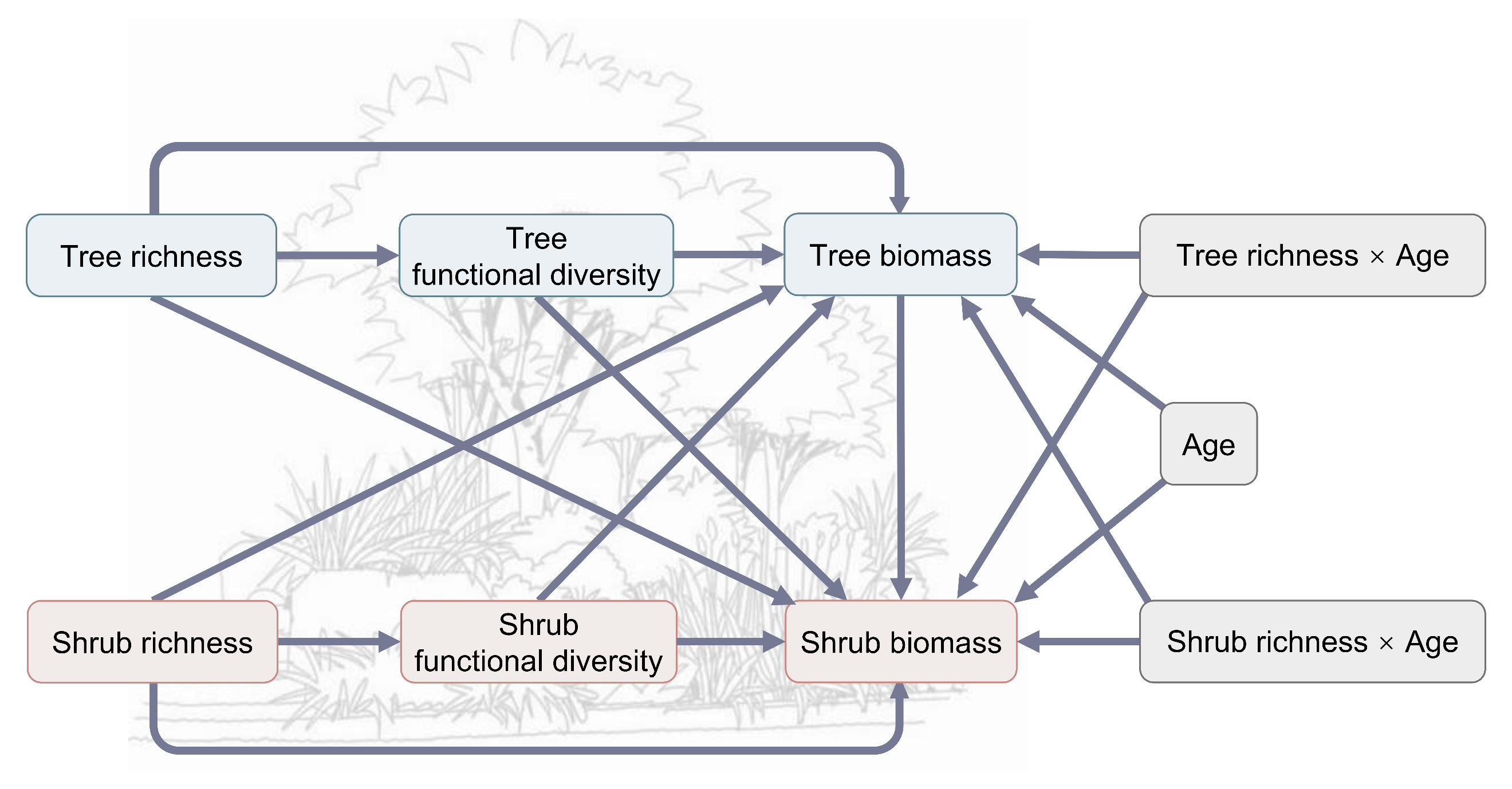


## Figure S4. Conceptual diagram for the relationships among tree and shrub species richness, functional diversity, biomass, and stand age. This conceptual diagram was based on the hypothesized paths and other plausible relationships.
